# Deciphering the metabolic perturbation in hepatic alveolar echinococcosis: a ^1^H NMR-based metabolomics study

**DOI:** 10.1186/s13071-019-3554-0

**Published:** 2019-06-13

**Authors:** Caigui Lin, Zhong Chen, Lingqiang Zhang, Zhiliang Wei, Kian-Kai Cheng, Yueyue Liu, Guiping Shen, Haining Fan, Jiyang Dong

**Affiliations:** 1 Department of Electronic Science, Fujian Provincial Key Laboratory for Plasma and Magnetic Resonance, Xiamen University, Xiamen, 361005 China; 2 Department of Hepatopancreatobiliary Surgery, Affiliated Hospital of Qinghai University, Xining, 810001 China; 3 Qinghai Province Key Laboratory of Hydatid Disease Research, Xining, 810001 China; 4 Department of Radiology, Johns Hopkins University, Baltimore, MA 21205 USA; 5 Innovation Centre in Agritechnology, Universiti Teknologi Malaysia, 84600 Muar, Johor Malaysia

**Keywords:** NMR, Metabolomics, Echinococcosis

## Abstract

**Background:**

Hepatic alveolar echinococcosis (HAE) is caused by the growth of *Echinococcus multilocularis* larvae in the liver. It is a chronic and potentially lethal parasitic disease. Early stage diagnosis for this disease is currently not available due to its long asymptomatic incubation period. In this study, a proton nuclear magnetic resonance (^1^H NMR)-based metabolomics approach was applied in conjunction with multivariate statistical analysis to investigate the altered metabolic profiles in blood serum and urine samples obtained from HAE patients. The aim of the study was to identify the metabolic signatures associated with HAE.

**Results:**

A total of 21 distinct metabolic differences between HAE patients and healthy individuals were identified, and they are associated with perturbations in amino acid metabolism, energy metabolism, glyoxylate and dicarboxylate metabolism. Furthermore, the present results showed that the Fischer ratio, which is the molar ratio of branched-chain amino acids to aromatic amino acids, was significantly lower (*P* < 0.001) in the blood serum obtained from the HAE patients than it was in the healthy patient group.

**Conclusions:**

The altered Fischer ratio, together with perturbations in metabolic pathways identified in the present study, may provide new insights into the mechanistic understanding of HAE pathogenesis and potential therapeutic interventions.

**Electronic supplementary material:**

The online version of this article (10.1186/s13071-019-3554-0) contains supplementary material, which is available to authorized users.

## Background

Echinococcosis, or hydatid disease, is a near-cosmopolitan zoonotic parasitic infection caused by the larval stage of pathogenic cestode parasites in the genus *Echinococcus* [1]. Different species of *Echinococcus* cause different diseases. The main types of echinococcosis include cystic echinococcosis (CE) and alveolar echinococcosis (AE), which are caused by *Echinococcus granulosus* and *Echinococcus multilocularis*, respectively [2]. Compared with CE, AE is associated with a lower occurrence, but higher lethality, if not treated with timely and proper treatments [3].

AE has been reported in its definitive hosts (canids) as well as in humans [4]. In humans, AE affects the liver in approximately 95% of diagnosed cases and is known as hepatic alveolar echinococcosis (HAE) [5]. HAE leads to liver-tissue injury or hepatic failure primarily through an infiltrative behaviour. It resembles the infiltrative proliferation of tumour growth and is clinically known as “worm cancer” or “parasite liver cancer”. After a long period of latent and asymptomatic stage, HAE can progress into a cirrhotic stage [6], or it can metastasize to other organs (e.g. the lungs and brain) [7–9] and cause local organ-function impairment and metastatic infiltration [10].

HAE is endemic to the northern hemisphere, including North America (Canada [11]), central Europe (France [12], Germany [13], Austria [14], Poland [15]), and central Asia (northern Iran [16], Mongolia [17] and western China [18]). Its global occurrence is mainly attributed to chronic anthropogenic influences, including increased globalization of animals and animal products, and altered human-animal interactions [4].

HAE patients often suffer from symptoms including fatigue, abdominal pain, hepatomegaly, nausea and vomiting. Notably, its long asymptomatic period of the early infection stage leads to difficulty in determining the time-point or place of infection [19]. In general, an HAE diagnosis is based on the integrated information including medical history, contact history with potential livestock hosts, clinical and pathologic findings, imaging examination, nucleic acid detection and serologic tests [11]. Modern imaging techniques, e.g. ultrasonography (US), magnetic resonance imaging (MRI), fluorodeoxyglucose positron emission tomography (FDG-PET), and computed tomography (CT), have been applied for the clinical diagnosis of HAE disease and have been used for long-term follow-up after therapeutic interventions [20]. However, these imaging methods have several limitations: CT is unable to delineate disease-induced perihepatic extension; MRI provides good definition of hydatid lesions but fails to assess hydatid fertility (viability); and FDG-PET provides non-invasive and accurate evaluation of metabolic activity in HAE at the complexity as well as the high expense of administering a radiotracer synthesised with an on-site cyclotron. Additionally, the effective diagnosis of early stage HAE remains difficult even with these advanced imaging techniques. This situation leads to late diagnosis at the middle or advanced stages, causing the patients not to undergo timely curative resection [21]. Thus, an effective early diagnosis and treatment for HAE may help to prevent complications, reduce postoperative reoccurrence, and improve the recovery rate and prognosis.

In the last decade, metabolomics approaches have been broadly used to study various liver diseases to determine potential early biomarkers and perturbed metabolic pathways [22]. However, there are limited reports on the applications of the proton nuclear magnetic resonance (^1^H NMR)-based metabolomics method on echinococcosis. In the present study, we used high-resolution ^1^H NMR based metabolomics and multivariate statistical analyses to (i) determine distinct metabolic patterns to differentiate HAE patients from healthy individuals and establish a metabolic fingerprint of biofluids obtained from HAE patients; (ii) provide some clues for the research on the molecular mechanism of HAE disease; (iii) demonstrate the feasibility and effectiveness of using metabolomics in echinococcosis studies; and (iv) provide a diagnostic reference for HAE patients.

## Methods

### Recruitment of participants

The recruited HAE patients were diagnosed at the Department of Hepatopancreatobiliary Surgery of the Affiliated Hospital of Qinghai University between July and September 2016. The diagnoses of HAE relied on the integrated use of imaging examination, serologic test, nucleic acid detection and pathologic observation (Additional file 1: Figure S1). In addition, patients’ medical history and contact with potential animal host of the disease were also taken into consideration. All HAE patients were classified according to the WHO/IWGE classification [23]: P1, peripheral lesions without proximal vascular and/or biliary involvement; P2, central lesions with proximal vascular and/or biliary involvement of one lobe; P3, central lesions with hilar vascular or biliary involvement of both lobes and/or with involvement of two hepatic veins; P4, any liver lesion with extension along the vessels and the biliary tree; N0, no regional involvement; N1, regional involvement of contiguous organs or tissues; M0, no metastasis; M1, metastasis.

To prevent confounding the metabolic effect from other diseases, patients with the following diseases were excluded from the study: diabetes, nephrosis, autoimmunity disease, malignant hepatic tumour, severe hepatorenal dysfunction, and post-transplantation immunoreaction. Prior to this study, no patient underwent surgery or received anti-inflammatory drugs (e.g. non-steroidal anti-inflammatory drugs and corticosteroids). Healthy volunteers from the patients’ families were recruited as the control group.

The sample size used in this study was estimated based on the prior power test with some knowledge from our previous preliminary study. The power analysis was performed using MetaboAnalyst v.4.0 software (http://www.metaboanalyst.ca) [24, 25], and the result indicated that a sample size of 18 was adequate to provide sufficiently high statistical power (Additional file 2: Figure S2).

### Sample collection and preparation

All blood and urine samples were collected in the morning, 12 h after the last meal of the previous day (fasting conditions). Blood samples were collected into tubes without anticoagulant and centrifuged at 6700×*g* at 4 °C for 15 min to obtain blood serum. The first morning urine samples were collected and centrifuged at 8500×*g* at 4 °C for 15 min and the supernatants were transferred into tubes. The blood serum and urine samples were aliquoted, snap-frozen in liquid nitrogen and stored at − 80 °C until further analysis.

Prior to analysis, an aliquot of 400 μl of blood serum was mixed with 200 μl of phosphate buffer solution (90 mM K_2_HPO_4_/NaH_2_PO_4_, pH 7.4, 0.9% NaCl, 99.9% D_2_O). Additionally, 300 μl of urine samples were mixed with a different phosphate buffer solution (300 μl, 1.5 M K_2_HPO_4_/NaH_2_PO_4_, pH 7.4, 99.9% D_2_O containing 0.3 mM 3-trimethylsilyl-propionic-2,2,3,3-d4 acid (TSP) as a chemical-shift reference for 0 ppm). D_2_O was used to provide the NMR spectrometer with a field frequency for locking. Buffered serum and urine samples were then centrifuged at 6700×*g* at 4 °C for 10 min to remove debris, and 500 μl of supernatant from each 600 μl mixture was transferred to 5-mm NMR tubes. In total, 36 serum and urine samples in NMR tubes were prepared and stored at 4 °C before NMR analysis.

## ^1^H-NMR experimentations

^1^H NMR experiments were performed using a Bruker NMR system (Bruker Biospin, Karlsruhe, Germany) operating at the proton frequency of 600 MHz. The operating temperature was set at 298 K. The Carr–Purcell–Meiboom–Gill (CPMG) sequence (waiting time ~ π/2 ~ [τ ~ π ~ τ]_n_ ~ acquisition) was used to acquire spectra of blood serum samples with an echo time (τ) of 250 μs and a free relaxation duration (2nτ) of 100 ms. For urine samples, nuclear overhauser effect spectroscopy (NOESY, waiting time ~ π/2 ~ t_1_ ~ π/2 ~ t_m_ ~ π/2 ~ acquisition) was implemented with a 2 s water suppression and mixing time (t_m_) of 120 ms. Free induction decays (FIDs) were recorded with 64 scans at a spectral width of 10 kHz. The FIDs were zero-padding to 32 k data points prior to fast Fourier transformation.

### Data processing of ^1^H-NMR spectra

Data pre-processing for the acquired ^1^H NMR spectra (including Fourier transformation, baseline correction and phase correction) was performed using MestReNova v.8.1.2 software (Mestrelab Research S.L., La Coruña, Spain). For peak alignment, the TSP signal was set as δ 0.00 for urine samples, and left split of the doublet of lactate signals was set as δ 1.336 for serum samples. Residual water signals (serum: δ 4.65–5.15; urine: δ 4.75–5.15), urea resonances (δ 5.70–6.40) and peak-free regions were selectively excluded for further analyses. The remaining spectra over the ranges of δ 0.8–8.5 for blood serum and δ 0.8–9.5 for urine were segmented into bucketed data using self-adaptive integration [26], and the results were exported as Microsoft Excel files. The data were normalized using the probabilistic quotient normalization (PQN) [27] method to compensate for overall concentration variations. The peaks in the acquired ^1^H NMR spectra were assigned based on previously published studies [28, 29], the KEGG database [30] and the HMDB database [31]. The relative concentrations for all assigned metabolites were evaluated based on their normalized peak integral area. For metabolites that gave rise to multiple peaks, those in the least overlapping spectral region were chosen for quantification.

### Multivariate and univariate statistical data analyses

Multivariate analyses of the pre-processed data were performed using SIMCA v.14.1 software (Umetrics, Umeå, Sweden). The data were examined by the non-supervised principal components analysis (PCA, unit variance scaling) and orthogonal partial least squares-discrimination analysis (OPLS-DA, unit variance scaling). A seven-fold cross-validation (i.e. 6/7 samples were used as training data and the remaining 1/7 samples as validation data) and permutation test (200 permutations) were performed and the obtained values of *R*^2^ (total explained variation) and *Q*^2^ (model predictability) were applied to validate the constructed models [32, 33]. An additional CV-ANOVA analysis was performed and the obtained *P*-value provided another venue of further model validation. The variable importance projection (VIP) and absolute correlation coefficient (|*r*|) calculated based on the OPLS-DA analysis were used as parameters for differential selection of metabolites. The VIP parameter, which was denoted as a unitless number, delineates the contribution of each predictor variable to the model and presents the influence of each predictor on the response variables. A larger VIP corresponds to a greater discriminatory power for the metabolites. In addition, metabolite sets were also analysed using Student’s t-test, which constitutes a simple statistical analysis to determine statistically significant metabolic variations in univariate analysis with the transformed *P*-value [34]. For a particular metabolite, its fold change was calculated based on the ratio of average concentrations between the HAE group and the control group.

In the present study, the results of multivariate statistical analysis were visualized and integrated with volcano plots to identify metabolites with significant difference between the HAE and control groups [35, 36]. In particular, the interactive volcano plot denotes − log_10_ (*P*-value) on the vertical axis and log_2_ (fold change) on the horizontal axis with circles of different sizes and colours for displaying VIP and |*r*| values, respectively.

Additionally, a *post-hoc* power analysis with specified significance level *α*, sample size, and effect size was executed with the online statistics software G*power v.3.1 (http://www.gpower.hhu.de/) [37, 38] to demonstrate the statistical power of the reported results. Moreover, receiver operator characteristic (ROC) curve analysis was applied to the resulting differential metabolites in HAE patients on Metaboanalyst v.4.0 (http://www.metaboanalyst.ca/) [39]. The area under the curve (AUC) of ROC was used to determine the specificity and the sensitivity of altered metabolites to discriminate HAE patients from healthy individuals.

## Results

### Clinical characteristics of subjects

The metadata of the participants (including age, gender, diameters of lesions, PNM classification and liver function test results) are summarized in Table 1. Comparing the control and the HAE groups, the age and gender of the participants showed no significant difference (*P* > 0.05). All HAE patients were classified into six categories according to the PNM classification by WHO/IWGE, as stated in methods. Among the participants, patients in the P3N1M1 and P4N1M1 classes showed lung metastasis.

**Table 1:** Clinical characteristics of healthy controls and HAE patients

Parameter	HAE group		Control group	
	*n* (%)	Mean ± SD	*n* (%)	Mean ± SD
Age (years)	18	32 ± 12	18	34 ± 12
Male	9 (50.0)	–	12 (66.7)	–
Female	9 (50.0)	–	6 (33.3)	–
BMI (kg/m^2^)	18	20.80 ± 2.96	18	22.00 ± 2.32
Lesion short diameter (cm)	18	10.09 ± 2.43	–	–
Lesion long diameter (cm)	18	12.74 ± 3.41	–	–
P1N0M0	2 (11.1)	–	–	–
P2N0M0	8 (44.4)	–	–	–
P2N1M0	5 (27.8)	–	–	–
P3N1M1	1 (5.6)	–	–	–
P4N1M0	1 (5.6)	–	–	–
P4N1M1	1 (5.6)	–	–	–
WBC (×10^9^/l)	18	7.80 ± 3.69	–	–
PLT (×10^9^/l)	18	296.06 ± 125.73	–	–
HB (g/l)	18	119.87 ± 33.13	–	–
PT (s)	18	13.20 ± 3.16	–	–
TB (µmol/l)	18	46.03 ± 49.06	–	–
ALT (U/l)	18	49.17 ± 58.40	–	–
ALP (U/l)	18	349.26 ± 332.27	–	–
ALB (g/l)	18	33.35 ± 4.04	–	–

*Abbreviations*: BMI, body mass index; PNM, parasite location within the liver, neighbouring organ involvement, metastases; WBC, white blood cells; PLT, blood platelets; HB, haemoglobin; PT, prothrombin time; TB, total bilirubin; ALT, alanine aminotransferase; ALP, alkaline phosphatase; ALB, albumin; SD, standard deviation

## ^1^H-NMR spectra of biological samples

**Fig. 1:**
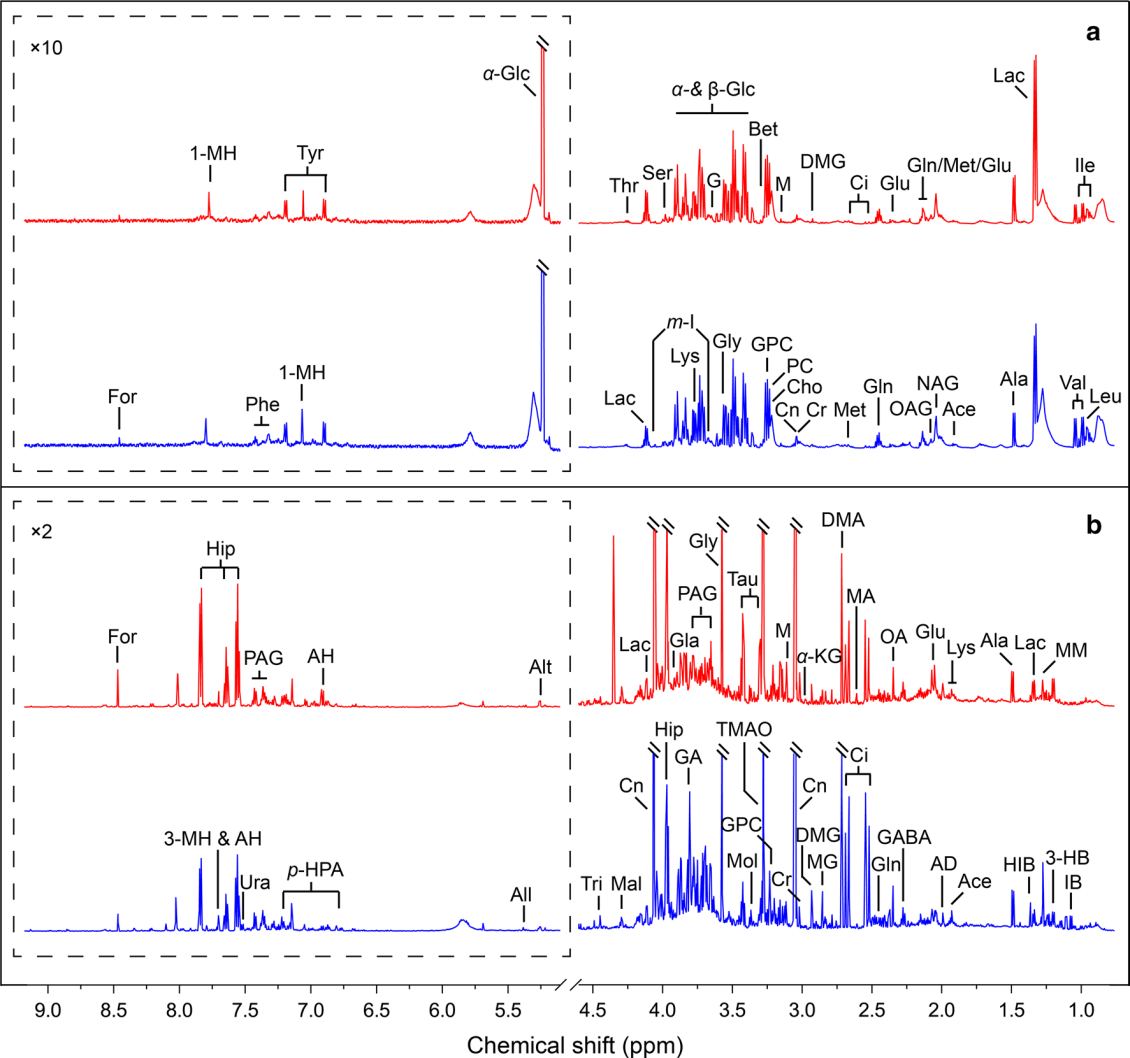
Resonance assignments of representative ^1^H NMR spectra at 600 MHz. **a** Serum and **b** urine samples, HAE (red line) and control (blue line) groups. *Abbreviations*: 1-MH, 1-methylhistidine; 3-HB, 3-hydroxybutyrate; 3-MH, 3-methylhistidine; Ace, acetate; AD, acetamide; AH, aminohippurate; Ala, alanine; All, allantoin; Alt, allantoate; Bet, betaine; Cho, choline; Ci, citrate; Cn, creatinine; Cr, creatine; DMA, dimethylamine; DMG, *N*,*N*-dimethylglycine; For, formate; G, glycerol; GA, guanidoacetate; GABA, *γ*-aminobutyrate; Gla, glycolate; Gln, glutamine; Glu, glutamate; Gly, glycine; GPC, glycerophosphocholine; HIB, 2-hydroxyisobutyrate; Hip, hippurate; IB, isobutyrate; Ile, isoleucine; Lac, lactate; Leu, leucine; Lys, lysine; M, malonate; MA, methylamine; Mal, malate; Met, methionine; MG, methylguanidine; *m*-I, *myo*-inositol; MM, methylmalonate; Mol, methanol; NAG, *N*-acetylglutamate; OA, oxaloacetate; OAG, *O*-acetylglycoprotein; PAG, phenylacetylglycine; PC, phosphocholine; Phe, phenylalanine; *p*-HPA, *para*-hydroxyphenylacetate; Ser, serine; Tau, taurine; Thr, threonine; TMAO, trimethylamine *N*-oxide; Tri, trigonelline; Tyr, tyrosine; Ura, uracil; Val, valine; *α*-Glc, *α*-glucose; *α*-KG, *α*-ketoglutarate; *β*-Glc, *β*-glucose

Typical ^1^H NMR spectra of biofluids collected from the HAE patients and healthy volunteers are shown in Fig. 1. In general, several metabolite classes were detected in the serum and urine samples, including metabolites involved in energy metabolism, neurotransmission, membrane metabolism and osmoregulation. In addition, serum metabolome comprised of a few glycoproteins, while a number of gut microbiota-related metabolites were detected in urine samples.

### Pattern recognition analysis

**Fig. 2:**
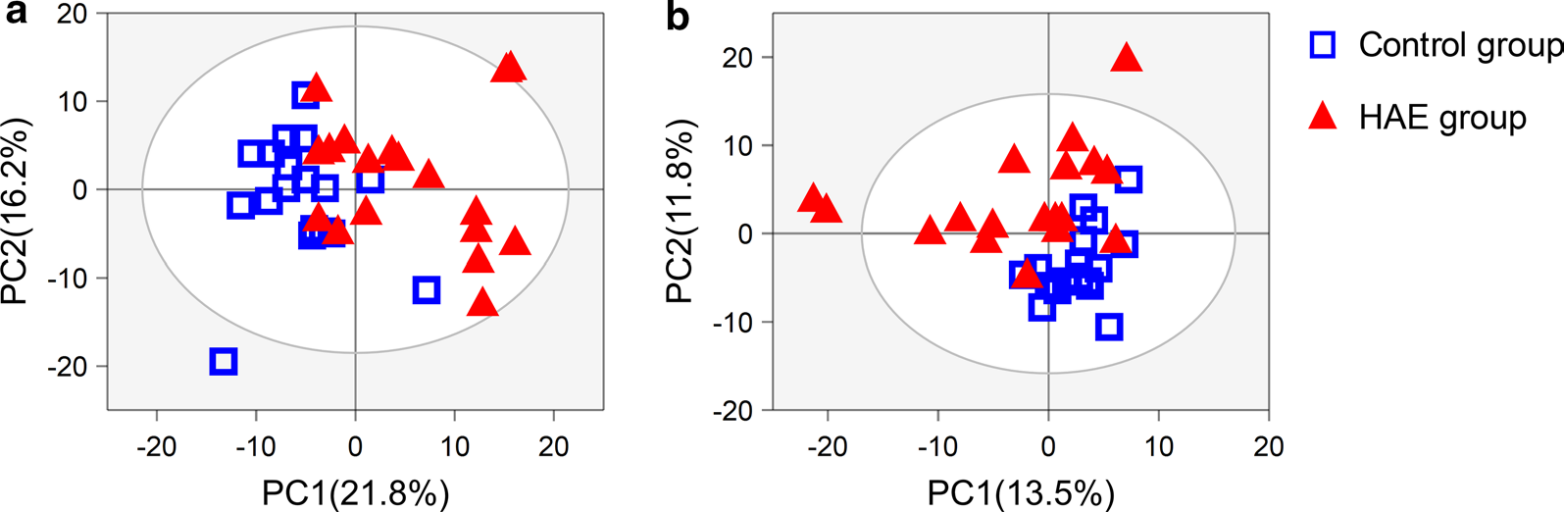
PCA score plots of control and HAE groups. **a** Serum and **b** urine samples. Each data point represents one subject. Two components accounted for 95.5% of total variances in the serum samples and 61.1% of total variances in the urine samples. The outer ellipse represents the 95% confidence interval (T^2^ Hotelling)

**Fig. 3:**
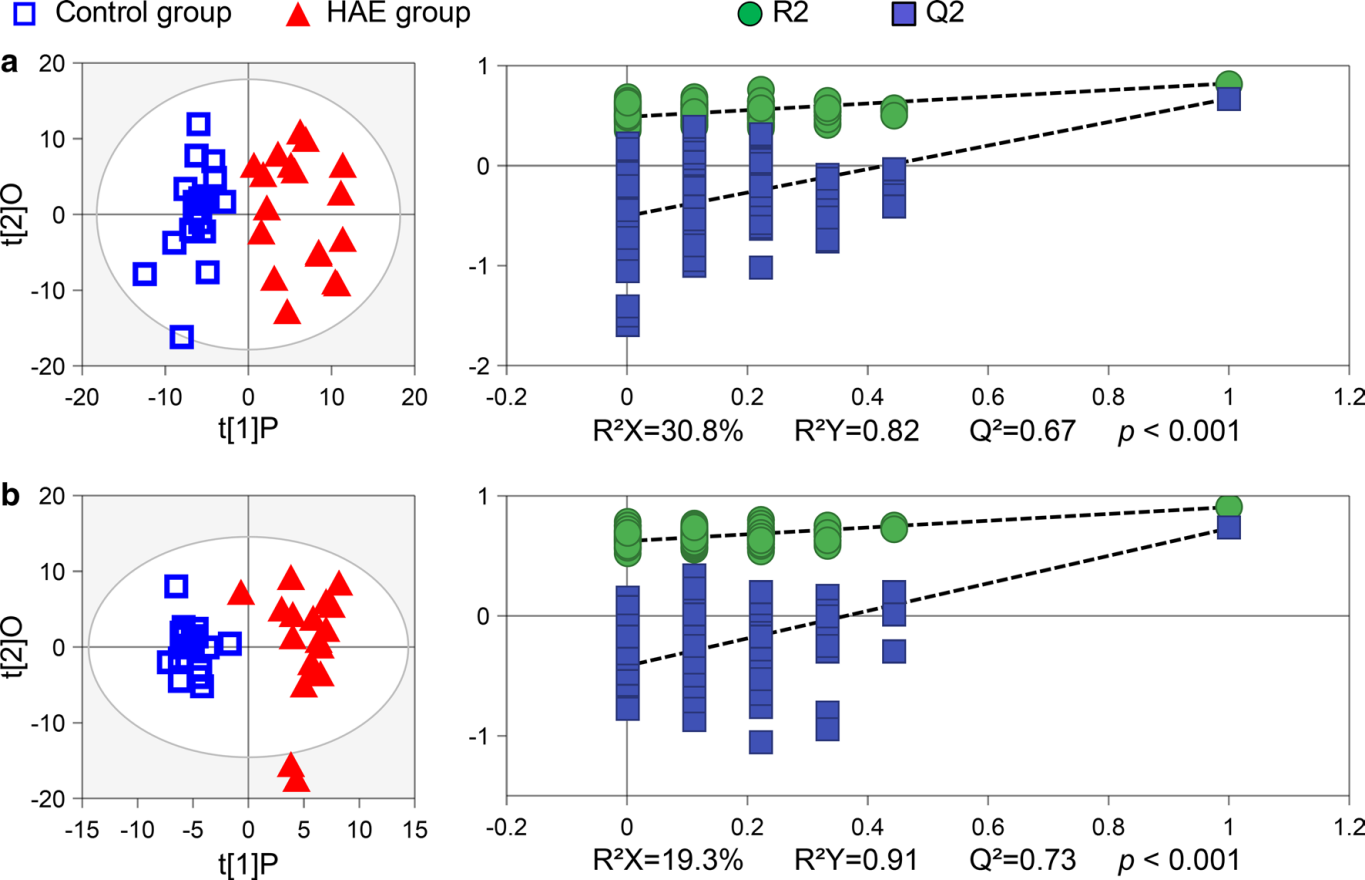
OPLS-DA scores plots (left panel) and their corresponding validation plots (right panel). **a** Serum and **b** urine samples. The *R*^2^ and *Q*^2^ values reflect the fraction of explained variance and model predictability, respectively

To identify HAE-induced metabolic changes, we performed multivariate analyses on the processed NMR data. First, the data were analysed by principal component analysis (PCA), of which the resulting score plots are shown in Fig. 2. Moderate groupings could be observed between the control and the HAE groups in the PCA score plots. In contrast, a distinct group separation could be achieved when the dataset was analysed using the supervised orthogonal partial least squares discrimination analysis (OPLS-DA). From the OPLS-DA score plots (Fig. 3), the control samples primarily distributed in the left hemisphere, while the HAE group clustered in the right hemisphere. The group separation indicated that HAE induced distinct metabolic changes in human serum and urine. The corresponding probability *P*-values by CV-ANOVA were also used to assess the statistical significance of metabolic differences between the control and the HAE groups. Moreover, the OPLS-DA models were found to be robust with good explained variations *R*^2^*Y* and predictive powers *Q*^2^ following permutation tests (200 permutations).

### Determination of differentiating metabolites for HAE

**Fig. 4:**
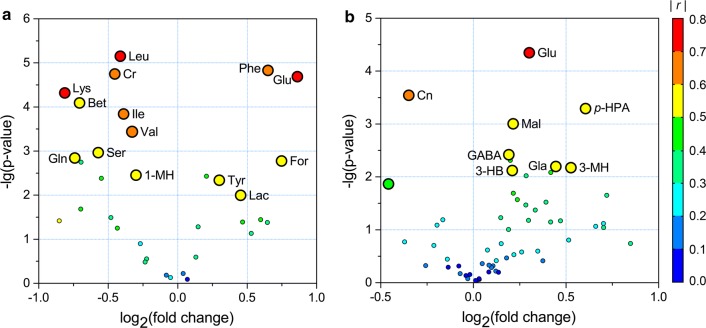
Enhanced volcano plots showing significantly changed metabolites. **a** Serum and **b** urine samples. Volcano plot shows − log_10_ (*P*-value) on the y-axis *versus* log_2_ (fold change) on the x-axis. Each point represents a different metabolite. The circles size and colour are determined based on the variable importance projection (VIP) and absolute correlation coefficient values (|*r*|), respectively. For each comparison, VIP values are categorized into two categories: top 30% and remaining 70%, with each represented by large and small circles, respectively. A warmer colour corresponds to higher |*r*|

To identify metabolic markers that differentiate the HAE patients from the healthy controls, we next presented the data using a four-dimensional enhanced volcano plot which may also offer intuitive data visualization [40], as shown in Fig. 4. In the volcano plot, the importance and significance of the metabolic changes were determined using the following criteria: variable importance projection (VIP) > top 30%, absolute correlation coefficient values (|*r*|) > 0.5, − log_10_ (*P*-value) > 2 (i.e. *P* < 0.01), and absolute log_2_ (fold change) > 0.20. Generally, identified metabolites with significant changes (those with a significant *P*-value, high fold change, VIP and |*r*|) by multivariate statistical analyses tended to locate at the upper-left or upper-right zones of the enhanced volcano plot with larger circle shapes and warmer colours, as marked with abbreviations in Fig. 4.

For blood serum samples, the volcano plot showed that HAE caused significant increases in the concentrations of phenylalanine (Phe), glutamate (Glu), tyrosine (Tyr), formate (For), and lactate (Lac), together with decreases of valine (Val), leucine (Leu), isoleucine (Ile), lysine (Lys), serine (Ser), glutamine (Gln), betaine (Bet), creatine (Cr) and 1-methylhistidine (1-MH) (Fig. 4a). Using a similar approach, urine samples from the HAE group were characterized by significantly higher levels of glutamate (Glu), malate (Mal), glycolate (Gla), 3-hydroxybutyrate (3-HB), *γ*-aminobutyrate (GABA), 3-methylhistidine (3-MH), and *p*-hydroxyphenylacetate (*p*-HPA), as well as lower concentration of creatinine (Cn) (Fig. 4b).

### Assessment of distinct metabolites for HAE

The statistical power analyses of the selected differential metabolites are presented in Table 2. First, the absolute difference between two independent means (the HAE and control groups) and the intra-group standard deviation were used to calculate the effect size. The effect size was considered to be large at a value of 0.80 according to the report by Cohen [41]. In addition, *post-hoc* analysis of the achieved statistical power calculation yielded an average value of 0.92 (SD = 0.09) under the given condition of significance level *α* = 0.05, sample size *n* = 18 and specified effective sizes (Additional file 3: Figure S3). The reliability and effectiveness of the present results were validated based on sufficiently high effect sizes and statistical power. Classic univariate ROC curve analyses of the total selected differential metabolites in serum and urine are listed in Table 3.

**Table 2:** *Post-hoc* power analyses of characteristic metabolites with G*power

Metabolite	Control group	HAE group	*t*-value^b^	*P*-value^c^	ES^d^	SP^e^
	CONC^a^	CONC^a^				
1-methylhistidine	15.52 ± 0.56	12.6 ± 0.74	3.38	3.55 × 10^−3^	1.05	0.86
3-hydroxybutyrate	11.51 ± 0.36	13.30 ± 0.52	3.03	7.56 × 10^−3^	0.95	0.79
3-methylhistidine	48.17 ± 1.64	69.37 ± 7.16	3.09	6.70 × 10^−3^	0.96	0.80
Betaine	10.90 ± 0.62	6.68 ± 0.83	4.61	2.50 × 10^−4^	1.36	0.98
Creatine	52.02 ± 1.19	39.87 ± 2.13	5.89	1.78 × 10^−5^	1.66	1.00
Creatinine	1438.09 ± 45.22	1128.99 ± 61.68	4.54	2.87 × 10^−4^	1.35	0.98
Formate	1.31 ± 0.10	2.20 ± 0.24	3.72	1.69 × 10^−3^	1.14	0.91
Glutamate	11.39 ± 1.29	20.71 ± 1.37	5.82	2.05 × 10^−5^	1.65	1.00
Glutamine	2.96 ± 0.35	1.77 ± 0.24	3.78	1.48 × 10^−3^	0.93	0.78
Glycolate	36.33 ± 1.34	49.44 ± 4.30	3.11	6.32 × 10^−3^	0.97	0.81
Isoleucine	17.14 ± 0.63	13.08 ± 0.71	4.87	1.44 × 10^−4^	1.43	0.99
Lactate	102.96 ± 10.14	140.90 ± 9.54	2.89	1.01 × 10^−2^	0.91	0.75
Leucine	15.70 ± 0.56	11.79 ± 0.48	6.36	7.05 × 10^−6^	1.77	1.00
Lysine	4.33 ± 0.19	2.47 ± 0.35	5.40	4.80 × 10^−5^	1.55	0.99
Malate	18.06 ± 0.33	20.95 ± 0.73	3.97	9.94 × 10^−4^	1.20	0.94
Phenylalanine	6.67 ± 0.61	10.02 ± 0.57	5.98	1.48 × 10^−5^	1.14	0.91
*p*-hydroxyphenylacetate	2.57 ± 0.08	3.91 ± 0.34	4.27	5.13 × 10^−4^	1.28	0.96
Serine	11.19 ± 0.75	7.52 ± 0.70	3.93	1.09 × 10^−3^	1.19	0.93
Tyrosine	8.11 ± 0.33	9.98 ± 0.52	3.26	4.59 × 10^−3^	1.01	0.84
Valine	28.35 ± 1.23	19.90 ± 0.78	4.43	3.66 × 10^−4^	1.94	1.00
*γ*-aminobutyrate	29.29 ± 0.78	33.44 ± 1.08	3.35	3.80 × 10^−3^	1.04	0.68

^a^The relative concentration (percentage of normalized integrals, mean ± standard error)

^b^The calculated *t*-value in the Student’s t-test based on 17 degrees of freedom

^c^The calculated statistical significance in the Student’s *t*-test

^d^The calculated effect size with G*power

^e^The calculated statistical power calculated as a function of the critical significance level (*α* = 0.05), given sample size (*n* = 18), and obtained variable effect size in G*power

**Table 3:** ROC curve analyses of metabolites to identify HAE patients

Metabolites	Sensitivity (%)	Specificity (%)	AUC (95%)
Serum			
Glutamate	100.00	88.89	0.924 (0.802–1.000)
Valine	94.44	88.89	0.920 (0.804–1.000)
Leucine	77.78	88.89	0.920 (0.801–0.981)
Phenylalanine	88.89	94.44	0.914 (0.782–1.000)
Creatine	83.33	88.89	0.870 (0.731–0.981)
Isoleucine	83.33	72.22	0.852 (0.716–0.951)
Lysine	83.33	77.78	0.849 (0.665–0.974)
Formate	77.78	88.89	0.833 (0.676–0.960)
Betaine	77.78	83.33	0.824 (0.684–0.948)
Serine	83.33	77.78	0.824 (0.676–0.941)
1-methylhistidine	72.22	77.78	0.785 (0.627–0.914)
Tyrosine	83.33	72.22	0.782 (0.609–0.924)
Lactate	83.33	77.78	0.762 (0.590–0.911)
Glutamine	66.67	83.33	0.716 (0.532–0.873)
Urine			
Glutamate	77.78	77.78	0.864 (0.714–0.957)
Creatinine	83.33	88.89	0.816 (0.645–0.952)
*p-*hydroxyphenylacetate	72.22	88.89	0.815 (0.611–0.955)
3-hydroxybutyrate	77.78	72.22	0.793 (0.619–0.914)
Malate	72.22	83.33	0.781 (0.614–0.920)
Glycolate	66.67	77.78	0.772 (0.572–0.900)
*γ*-aminobutyrate	72.22	72.22	0.769 (0.586–0.903)
3-methylhistidine	72.22	77.78	0.765 (0.591–0.920)

**Fig. 5:**
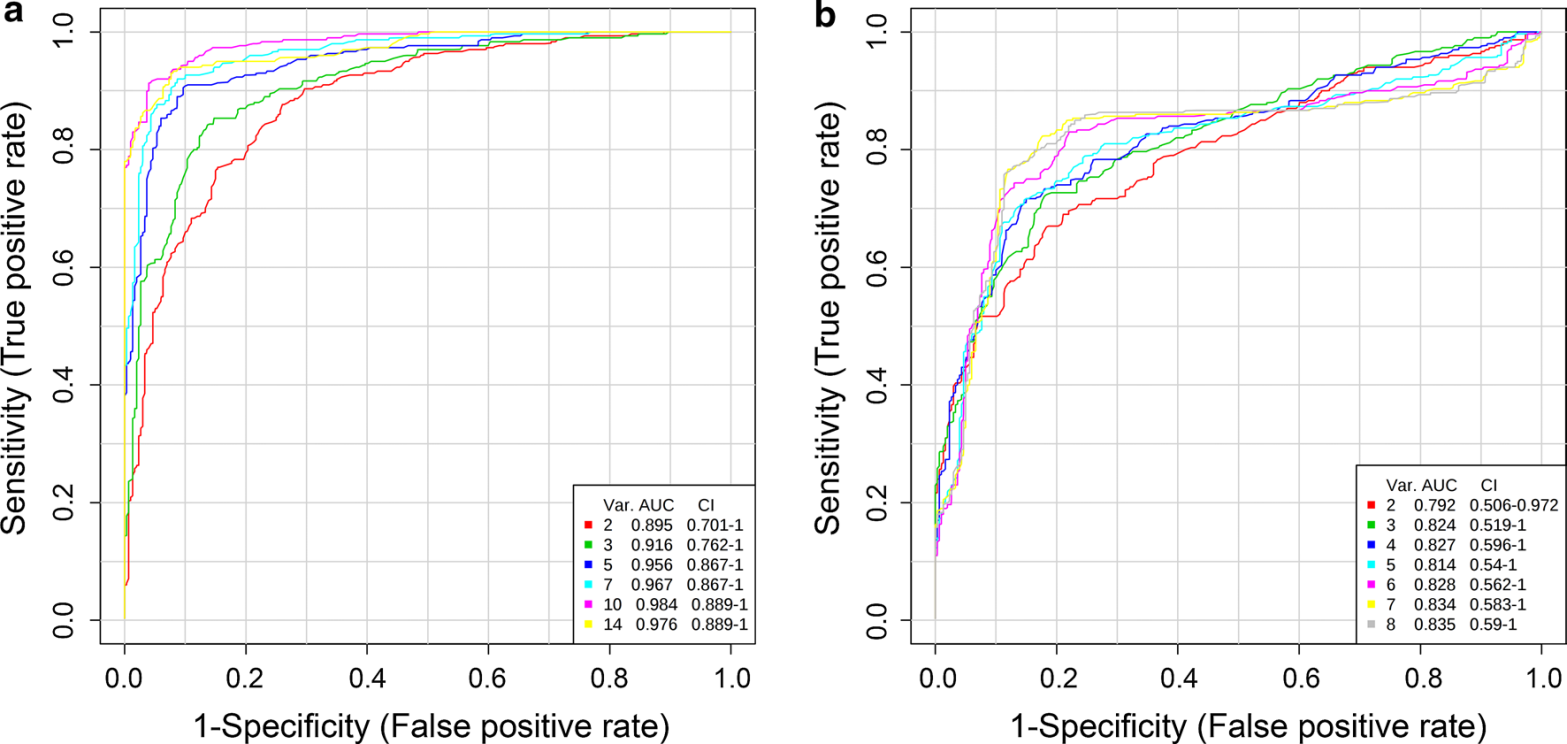
Multivariate ROC curve analyses of distinctive metabolites for discrimination of HAE patients from healthy individuals. Serum (**a**) and urine (**b**) samples. PLS-DA and univariate AUROC were used for classification and feature ranking methods, respectively. Var. (variables) indicates the number of selected metabolites

The sensitivity and specificity were evaluated for each potential metabolite with area under the curve (AUC) values. In principle, an AUC value close to 1 indicates an excellent diagnostic power, and an AUC of 0.5 suggests no diagnostic power. Therefore, metabolites with high AUC values have a better predictive power for identifying HAE patients. In serum samples, ROC curves showed that glutamate (AUC = 0.924), leucine (AUC = 0.920), valine (AUC = 0.920) and phenylalanine (AUC = 0.914) have a high discriminatory ability for HAE. Overall, 10 metabolites had an AUC > 0.8. In urine samples, glutamate, creatinine and *p*-hydroxyphenylacetate exhibited an AUC > 0.8 (Additional file 4: Figure S4 and Additional file 5: Figure S5). As individual metabolites may not be sufficient to predict the correct phenotype, we performed multivariate ROC curve analyses to investigate combinations of metabolites as potential differential metabolic signatures in serum and urine samples (Fig. 5). ROC curves were generated by Monte-Carlo cross validations. In each Monte-Carlo cross validation, two thirds of the samples were used to evaluate the feature importance. Top important features were used to build classification models, which were validated on the remaining one third of the samples. In serum samples, the best model consisting of the top 10 metabolites (glutamate, leucine, valine and phenylalanine, etc.) showed an AUC value of 0.984 (95% CI: 0.889–1), which did not increase further even when more metabolites were added into the analyses. The results suggest that the combination of the 10 differential metabolites has the potential of distinguishing the HAE patients from the healthy controls (Fig. 5a). In urine samples, metabolite combination increased the AUC value from 0.792 to 0.835 (Fig. 5b). These results suggested the potential roles of these differential metabolites in the HAE pathogenesis.

In addition, we used the 21 characteristic metabolites to build a PLS-DA model (Additional file 6: Figure S6) for HAE identification. The misclassification table of the HAE PLS-DA model with six-fold cross-validation suggests that the combination of 21 characteristic metabolites can serve as a good identifier for HAE samples in the present dataset (Table 4).

**Table 4:** Misclassification of the PLS-DA model comprising characteristic metabolites

Classes	Members	Correct (%)	Control group	HAE group
Control group	18	100	18	0
HAE group	18	100	0	18
Total	36	100	18	18
Fisher’s probability	< 0.001			

### Metabolic pathway analysis

**Fig. 6:**
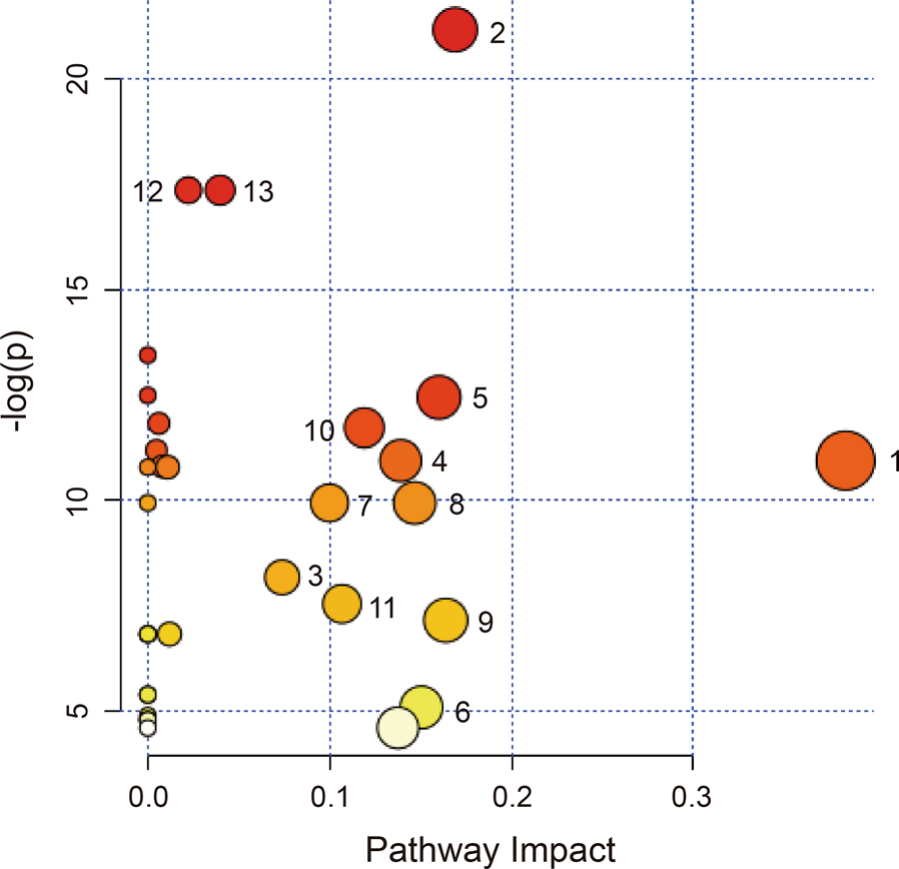
Bubble plots showing altered metabolic pathways perturbed by HAE. Bubble area is proportional to the impact of each pathway with colour denoting the significance from highest (in red) to lowest (in white). *Key*: 1, alanine, aspartate and glutamate metabolism; 2, aminoacyl-tRNA biosynthesis; 3, arginine and proline metabolism; 4, d-glutamine and d-glutamate metabolism; 5, glycine, serine and threonine metabolism; 6, glyoxylate and dicarboxylate metabolism; 7, lysine biosynthesis; 8, lysine degradation; 9, methane metabolism; 10, phenylalanine metabolism; 11, tyrosine metabolism; 12, valine, leucine and isoleucine biosynthesis; 13, valine, leucine and isoleucine degradation

Next, metabolic pathway analysis of the identified differential metabolites was performed using MetaboAnalyst v.4.0 software to investigate the most perturbed metabolic pathways in HAE. The analysis suggested perturbations in multiple metabolic pathways including alanine, aspartate and glutamate, arginine and proline metabolism, d-glutamine and d-glutamate metabolism, glycine, serine and threonine metabolism, glyoxylate and dicarboxylate metabolism, lysine metabolism, methane metabolism, phenylalanine metabolism, tyrosine metabolism and valine, leucine and isoleucine metabolism (Fig. 6). These pathways were selected based on an impact value ≥ 0.02 and − log(*P*) ≥ 5 and considered as the most relevant pathways in HAE (Table 5).

**Table 5:** Pathway analysis results obtained with MetaboAnalyst

Key	Pathway name	Total^a^	Hit^b^	Impact^c^	− log(*P*)
1	Alanine, aspartate and glutamate metabolism	24	2	0.38	10.94
2	Aminoacyl-tRNA biosynthesis	75	9	0.17	21.18
3	Arginine and proline metabolism	77	4	0.07	8.17
4	d-glutamine and d-glutamate metabolism	11	2	0.14	10.94
5	Glycine, serine and threonine metabolism	48	3	0.16	12.44
6	Glyoxylate and dicarboxylate metabolism	50	2	0.15	5.08
7	Lysine biosynthesis	32	1	0.10	9.94
8	Lysine degradation	47	1	0.15	9.94
9	Methane metabolism	34	2	0.16	7.15
10	Phenylalanine metabolism	45	3	0.12	11.72
11	Tyrosine metabolism	76	2	0.11	7.54
12	Valine, leucine and isoleucine biosynthesis	27	3	0.04	17.37
13	Valine, leucine and isoleucine degradation	40	3	0.02	17.37

^a^The total number of compounds in the pathway

^b^The actual matched number from the user uploaded data

^c^The pathway impact value calculated from pathway topology analysis

^d^The original *P*-value calculated from the logarithm analysis

## Discussion

**Fig. 7:**
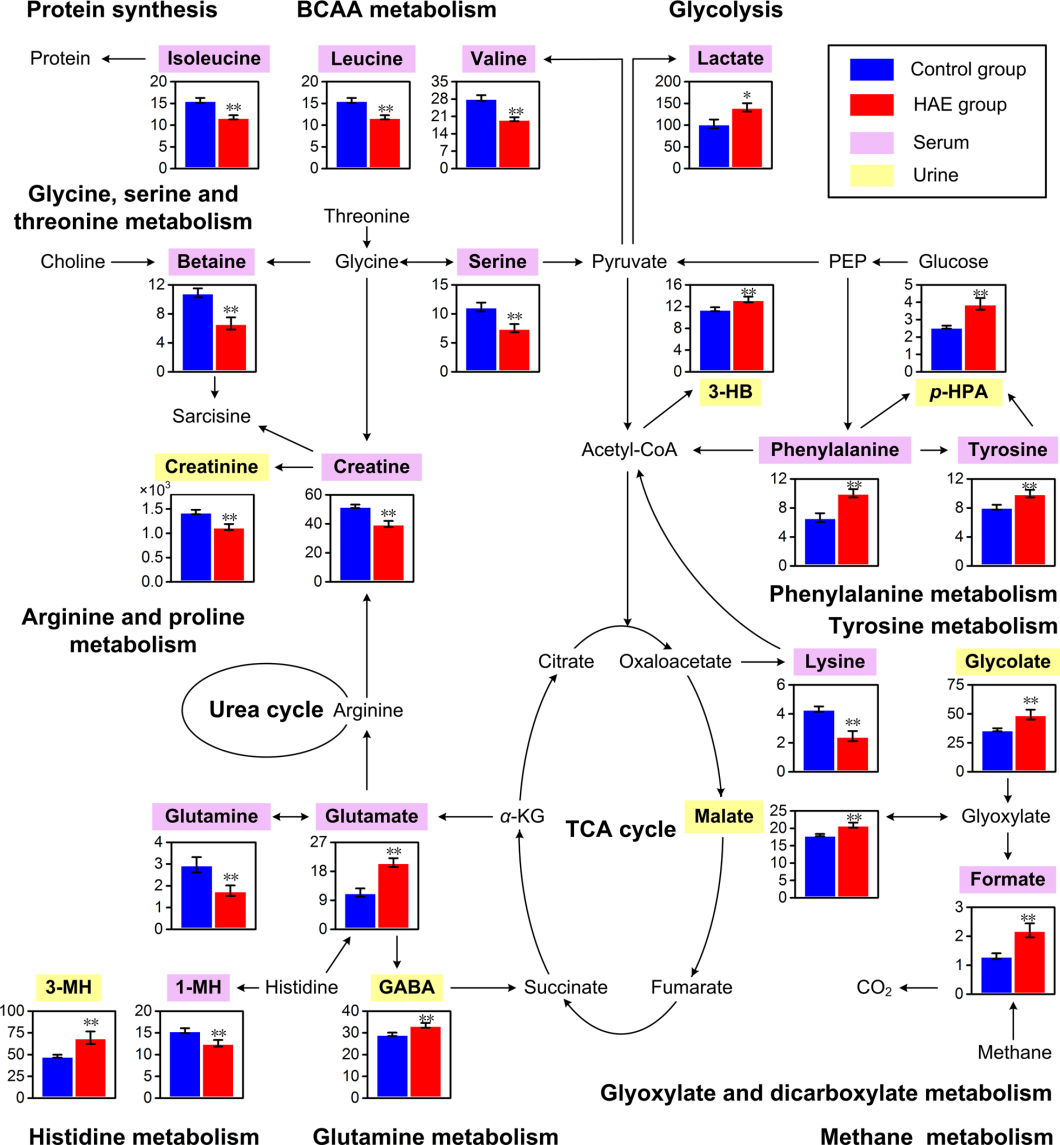
Mapping of differential metabolites induced by HAE onto metabolic pathways. Each bar graph represents one metabolite in relative concentration (mean ± standard error) of control (blue) and HAE (red) groups. *Indicates *P* < 0.05 statistical significance relative to control group; **Indicates *P* < 0.01 statistical significance relative to control group

The present study reveals metabolic perturbation in response to HAE disease using ^1^H NMR based metabolomics. First, ^1^H NMR spectra and pattern recognition analyses of serum and urine showed substantial changes in the human metabolome due to HAE. Secondly, a total number of 21 metabolites were identified that were able to discriminate the HAE patients from the healthy individuals. Thirdly, metabolic network analyses revealed HAE-induced modulations occur in several metabolic pathways, i.e. amino acid metabolism, energy metabolism, glyoxylate and dicarboxylate metabolism, and methane metabolism (Figs. 6, 7). These metabolic changes may provide new perspectives into the understanding of biological mechanisms that occur during HAE infection.

In the present study, HAE was found to result in significant changes in amino acid metabolism. This is evidenced by decreased levels in lysine, serine, glutamine and branched-chain amino acids (BCAA; i.e. valine, leucine and isoleucine), together with increased levels of glutamate and aromatic amino acids (AAA; e.g. tyrosine and phenylalanine) (Figs. 4, 7). Tyrosine is the first product in phenylalanine catabolism. Previously, it was reported that directionalities of the plasma-concentration shift of these two metabolites are the same and conversion of phenylalanine to tyrosine is an exclusive function of the liver [42, 43]. Thus, the parallel accumulations of AAA (i.e. tyrosine and phenylalanine) in blood serum suggested the possible loss of liver function during HAE infection. As essential amino acids, BCAA account for approximately 20% of our dietary protein intake and are key regulators of protein synthesis in animals and humans. BCAA disposal is tightly mediated by phosphorylation and dephosphorylation of the branched-chain *α*-keto acid dehydrogenase complex (BCKDC) [44]. The BCKDC is the rate-limiting enzyme in BCAA oxidative catabolism; the possible activation of BCKDC by HAE increases the BCAA catabolism to promote protein synthesis in case of increased protein degradation due to cell damage or necrosis during HAE infection. Moreover, the role of BCAA in the immune function has gained research interest in the last decade [45]. BCAA are oxidized by immune cells as fuel sources and incorporated as precursors for the synthesis of new immune cells, effector molecules and protective molecules [46]. The host immune response induced by HAE may lead to a higher consumption of BCAA to maintain immune function.

In addition, increased urinary concentrations of GABA and 3-methylhistidine were found in the HAE patients. GABA is known to have relaxant effects on muscle tone while 3-methylhistidine is an indicator of muscle catabolism [47]. Taken together, the data indicates that HAE may have metabolic effect on muscle function. In general, 3-methylhistidine is released during muscle-protein degradation and is not reutilized for muscle protein synthesis. This observation suggests that the immune response to HAE requires not only redirection of abundant energy but also metabolic resources from other tissues, especially from skeletal muscles. Hence, the consumption of BCAA in promoting protein synthesis may be due to muscle-protein synthesis. On the other hand, 98% of body creatine is found in skeletal muscle and a constant fraction of the body creatine pool is converted each day to creatinine. Hence, it has long been recognized that urinary creatinine excretion constitutes a good reflection of the skeletal muscle mass [48]. The decline of urine creatinine reflects a corresponding muscle mass loss in the HAE patients. The symptoms of fatigue and weight loss for HAE patients may be attributed to muscle mass loss and altered muscle function.

Being large neutral amino acids, BCAA and AAA can travel across the blood-brain barrier into the brain through the same type of neutral amino acid transporter LAT-1 [49]. Therefore, the raised AAA (tyrosine and phenylalanine) and reduced BCAA (valine, leucine and isoleucine) levels in the blood serum of the HAE patients may contribute to a subsequent increased influx of tyrosine and phenylalanine in the brain during HAE infection. However, the production of important bioamine neurotransmitters (i.e. dopamine, epinephrine and norepinephrine) is relatively insensitive to the cerebral levels of their precursors (i.e. tyrosine and phenylalanine). By contrast, the production of false neurotransmitters (e.g. octopamine or phenylethanolamine) can be stimulated by the cerebral elevated levels of tyrosine and phenylalanine, leading to an increased number of false neurotransmitters. These false neurotransmitters are structurally similar to “authentic” neurotransmitters, but they lack the ability to propagate neural excitation. This may result in imbalances of neurotransmitter synthesis. Homeostasis between excitatory (e.g. glutamate) and inhibitory (e.g. GABA) neurotransmitters is essential for maintaining the normal functioning of the central nervous system (CNS) [50]. In our study, elevations of excitatory glutamate and inhibitory GABA along with glutamine reduction indicated that neurotransmitter recycling disorder occurs in the CNS during HAE infection due to the imbalance between excitation and inhibition. Moreover, alterations of GABA and glutamate in the same direction have been reported to contribute to the pathophysiology of depression based on behavioural observations [51, 52].

Previously, Fischer et al. [53] reported decreased BCAA and increased AAA levels in the blood during hepatic failure in patients with liver disease. The ratio between the three BCAA (valine, leucine and isoleucine) and two AAA (tyrosine and phenylalanine) has been termed as the Fischer ratio for a quick assessment of liver disease. With a simultaneous consideration over variations of BCAA and AAA, the amino acid imbalance will lead to a lower Fischer ratio in cases of hepatic failures [54–56]. In this study, a decreased Fischer ratio of serum samples for the HAE group (*P* < 0.001) indicated that HAE may have triggered hepatic failure in these patients. Therefore, the Fischer ratio may emerge as a reliable index for diagnosing HAE. Although the present ^1^H NMR data do not provide absolute concentrations of AAA and BCAA, the Fischer ratio calculated using normalized data was found to provide good discrimination between the HAE patients and the healthy individuals.

Alveoli, host metastasis and massive immune response due to HAE are known to lead to the generation of a large amount of unstable reactive oxygen species (ROS) [11, 57]. Over-generation of ROS induces an oxidative-stress response with upregulation of the tricarboxylic acid (TCA) cycle and glycolysis to produce more ATP for cell maintenance and proliferation. In the present study, the increased levels of malate and lactate in the HAE group support the notion of enhanced TCA cycle and glycolysis. In addition, the present results also showed decreased creatine concentration in the HAE group, which provides further support for the perturbed energy metabolism in liver mitochondria during HAE infection. Creatine acts as an intracellular high-energy phosphate shuttle and plays a crucial role in maintaining cellular energy homeostasis. It can be catabolised to creatinine to provide immediate adequate levels of ATP in cases of energy deficit [58]. Thus, a decreased level of creatine in blood serum may reflect its consumption to alleviate HAE-induced energy deficit. Apart from being an ergogenic aid, creatine has a cytoprotective property as a direct radical scavenger against ROS [59]. Therefore, the lower concentration of creatine in the HAE group can partly be attributed to the antioxidant reactions during HAE infections.

It should be noted that the present study has several limitations. First, a larger number of participants are needed to further increase the reliability and accuracy of the present results. Secondly, an integrated application of NMR with LC–MS or GC–MS will extend the metabolite coverage, and multiple -omic technologies (e.g. genomics and proteomics) can cross-validate and better support the experimental results. Thirdly, data for early stage HAE are currently not available due to the limitation of sample number. Data from different stages of HAE are important for the early detection, prediction and diagnosis of HAE. Finally, a discriminating method that can distinguish HAE from other types of hepatic disease is not available in the present study. If developing such a method, it is crucial to prevent clinical misdiagnoses as HAE exhibits similarities (e.g. lower Fischer ratio) with other hepatic diseases. The possibility of using the Fischer ratio and a unique metabolic signature for HAE to distinguish between different hepatic diseases warrants further study.

The attractive properties of the NMR-based metabolomics approach, i.e. excellent repeatability, short detection duration and multiple metabolite coverage in a single measurement, makes it suitable for the HAE studies. A systematic exploration of multiple metabolites of small molecules helps to reveal the comprehensive metabolic variations induced by HAE infection. The early stage HAE diagnosis may be possible with a combination of both metabolomics and imaging techniques. For instance, chemical-exchange weighted magnetic resonance imaging techniques can be utilized to yield maps weighted by metabolites of interest [60–62]. The maps are obtained through an exchange effect with water and are characterized by significant signal enhancement to observe small metabolic variations unnoticeable with other imaging modalities. A general issue for these techniques is the lack of specificity, and modulations from adjacent resonances are inevitably and unfavourably introduced. NMR-based metabolomics with high specificity can thus be combined for improved analyses and better interpretation of the results.

## Conclusions

In this study, a ^1^H NMR-based metabolomics approach was used to investigate metabolic perturbations of HAE. Multivariate statistical analyses have highlighted several characteristic metabolites for HAE, e.g. decreased BCAA (valine, leucine and isoleucine), increased AAA (tyrosine and phenylalanine), increased lactate and decreased creatine, to list a few. The altered Fischer ratio together with specific metabolic changes identified in the present study may provide new insights into the molecular mechanism of HAE, and provide some clues to therapeutic intervention for HAE.

## Additional files

**Additional file 1: Figure S1.** Typical abdominal CT images of echinococcosis lesions in patients. Echinococcosis lesions are indicated by arrowheads. **a** Hepatic alveolar echinococcosis; CT image reveals dense calcification with irregular and indistinct margins in the lesion. **b** Hepatic cystic echinococcosis; CT image shows a sharply defined homogeneous cyst with multiple daughter cyst.

**Additional file 2: Figure S2.** Predicted power profile with sample size per group (false discovery rate of 0.005).

**Additional file 3: Figure S3.** The main window of the *post-hoc* power analysis specification in Gpower v.3.1 and the “effect size” drawer. Take as an example the metabolite of 1-methylhistidine which is different between control and HAE groups.

**Additional file 4: Figure S4.** Univariate ROC curve analyses of metabolites in serum for discrimination of HAE patients from healthy individuals.

**Additional file 5: Figure S5.** Univariate ROC curve analyses of metabolites in urine for discrimination of HAE patients from healthy individuals.

**Additional file 6: Figure S6.** PLS-DA scores plot (left panel) and permutation test (right panel) of PLS-DA model comprising 21 identified characteristic metabolites.

## Data Availability

The data supporting the findings of this article are included within the article and its additional files. The ^1^H NMR spectral raw data have been submitted to the MetaboLights repository under study identifier MTBLS981.

## Abbreviations

AAA: aromatic amino acids
AE: alveolar echinococcosis
AUC: area under the curve
BCAA: branched-chain amino acids
BCKDC: branched-chain *α*-keto acid dehydrogenase complex
CE: cystic echinococcosis
CNS: central nervous system
CT: computed tomography
CV-ANOVA: ANOVA of the cross-validated residuals
FDG-PET: fluorodeoxyglucose positron emission tomography
FID: free induction decays
GABA: *γ*-aminobutyrate
GC–MS: gas chromatography–mass spectrometry
HAE: hepatic alveolar echinococcosis
HMDB: human metabolome database
IWGE: informal working group on echinococcosis
KEGG: Kyoto Encyclopedia of Genes and Genomes
LC–MS: liquid chromatography–mass spectrometry
MRI: magnetic resonance imaging
NMR: nuclear magnetic resonance
OPLS-DA: orthogonal partial least squares-discrimination analysis
PCA: principal components analysis
PQN: probabilistic quotient normalization
ROC: receiver operator characteristic
ROS: reactive oxygen species
TCA: tricarboxylic acid
US: ultrasonography
VIP: variable importance projection
WHO: World Health Organization
