# Supplementary material for: Deciphering the metabolic perturbation in hepatic alveolar echinococcosis: a 1H NMR-based metabolomics study
Source: Parasit Vectors. 2019 Jun 13;12:300. doi: 10.1186/s13071-019-3554-0 (PMC6567409; doi:10.1186/s13071-019-3554-0)
Supplement: Supplementary file 1 — Additional file 1: Figure S1. Typical abdominal CT images of echinococcosis lesions in patients. Echinococcosis lesions are indicated by arrowheads. a Hepatic alveolar echinococcosis; CT image reveals dense calcification with irregular and indistinct margins in the lesion. b Hepatic cystic echinococcosis; CT image shows a sharply defined homogeneous cyst with multiple daughter cyst. [file 13071_2019_3554_MOESM1_ESM.pdf]

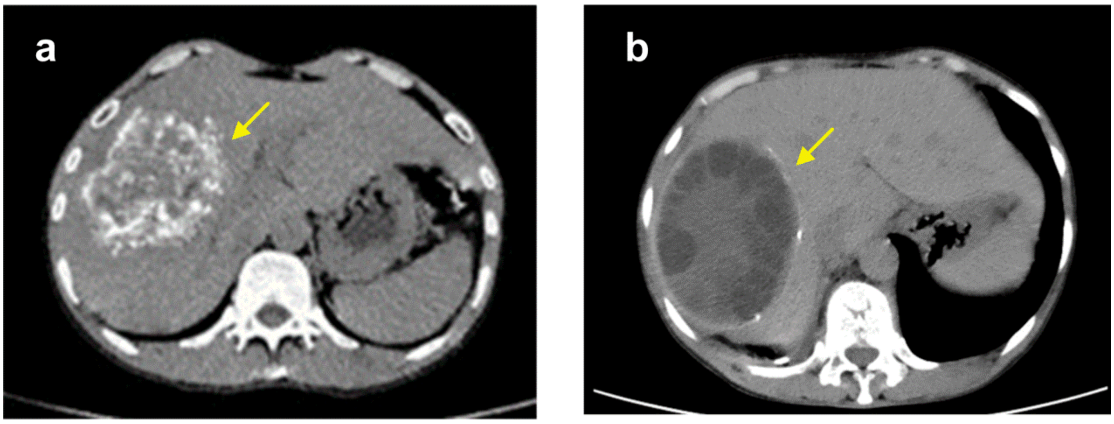

Additional file 1. Figure S1. Typical abdominal CT images of echinococcosis lesions in patients. Echinococcosis lesions are indicated by arrowheads, (a) hepatic alveolar echinococcosis, CT image reveals dense calcification with irregular and indistinct margins in the lesion; (b) hepatic cystic echinococcosis, CT image shows a sharply defined homogeneous cyst with multiple daughter cyst.
