# Supplementary material for: Deciphering the metabolic perturbation in hepatic alveolar echinococcosis: a 1H NMR-based metabolomics study
Source: Parasit Vectors. 2019 Jun 13;12:300. doi: 10.1186/s13071-019-3554-0 (PMC6567409; doi:10.1186/s13071-019-3554-0)
Supplement: Supplementary file 2 — Additional file 2: Figure S2. Predicted power profile with sample size per group (false discovery rate of 0.005). [file 13071_2019_3554_MOESM2_ESM.pdf]

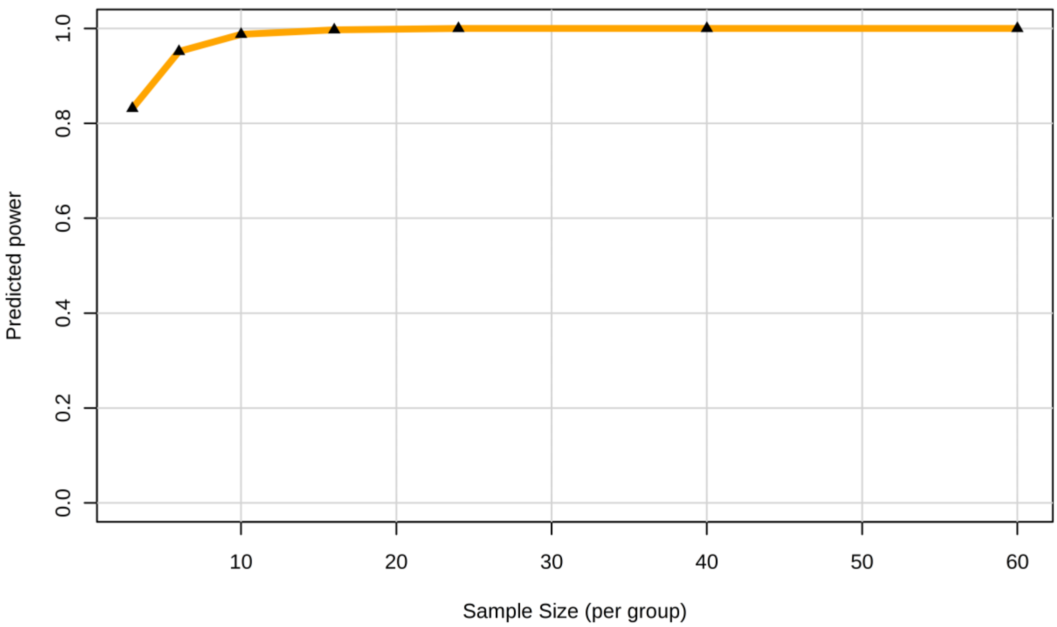

Additional file 2. Figure S2. Predicted power profile with sample size per group (false discovery rate of 0.005).
