# Supplementary material for: Deciphering the metabolic perturbation in hepatic alveolar echinococcosis: a 1H NMR-based metabolomics study
Source: Parasit Vectors. 2019 Jun 13;12:300. doi: 10.1186/s13071-019-3554-0 (PMC6567409; doi:10.1186/s13071-019-3554-0)
Supplement: Supplementary file 3 — Additional file 3: Figure S3. The main window of the post-hoc power analysis specification in Gpower v.3.1 and the “effect size” drawer. Take as an example the metabolite of 1-methylhistidine which is different between control and HAE groups. [file 13071_2019_3554_MOESM3_ESM.pdf]

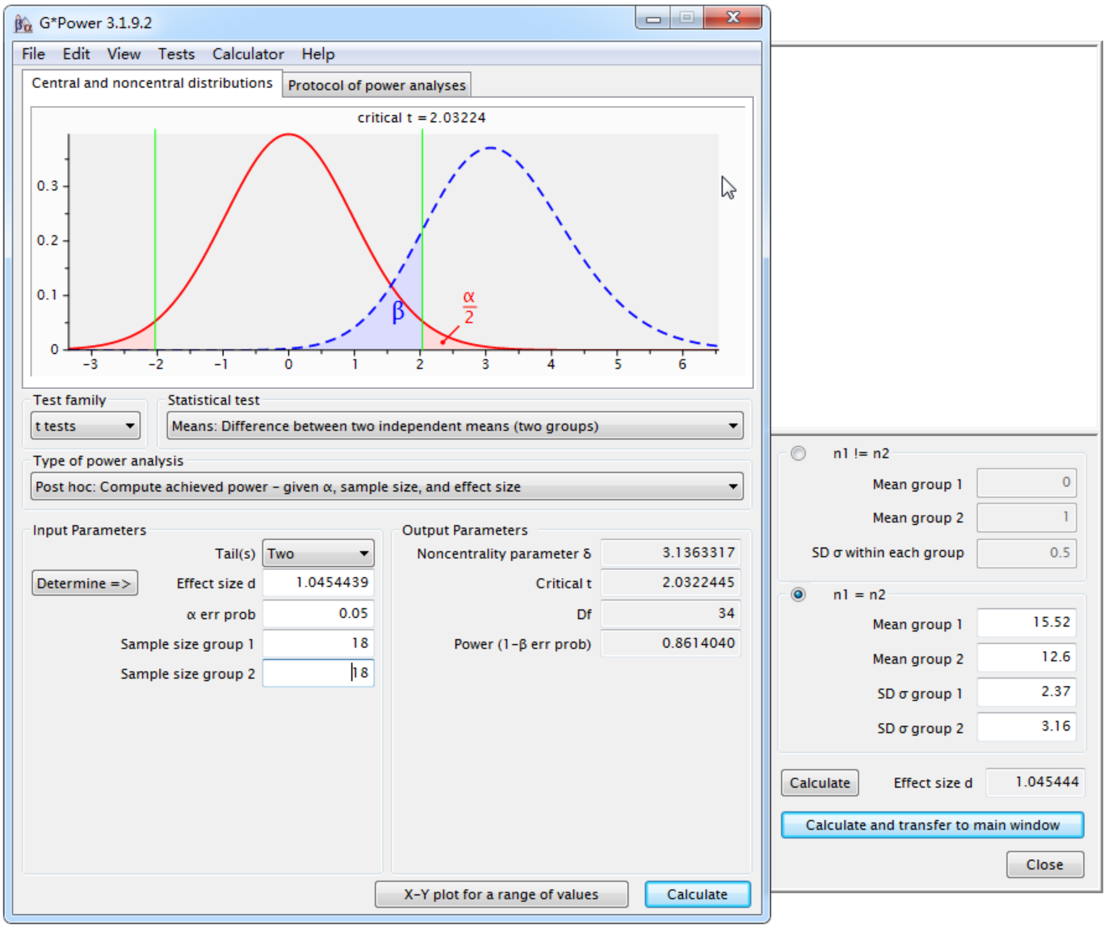

Additional file 3. Figure S3. The main window of the post hoc power analysis specification in Gpower 3.1 and the "effect size" drawer. Take the 1-methylhistidine selected between the control and HAE groups as an example.
