# Supplementary material for: Deciphering the metabolic perturbation in hepatic alveolar echinococcosis: a 1H NMR-based metabolomics study
Source: Parasit Vectors. 2019 Jun 13;12:300. doi: 10.1186/s13071-019-3554-0 (PMC6567409; doi:10.1186/s13071-019-3554-0)
Supplement: Supplementary file 4 — Additional file 4: Figure S4. Univariate ROC curve analyses of metabolites in serum for discrimination of HAE patients from healthy individuals. [file 13071_2019_3554_MOESM4_ESM.pdf]

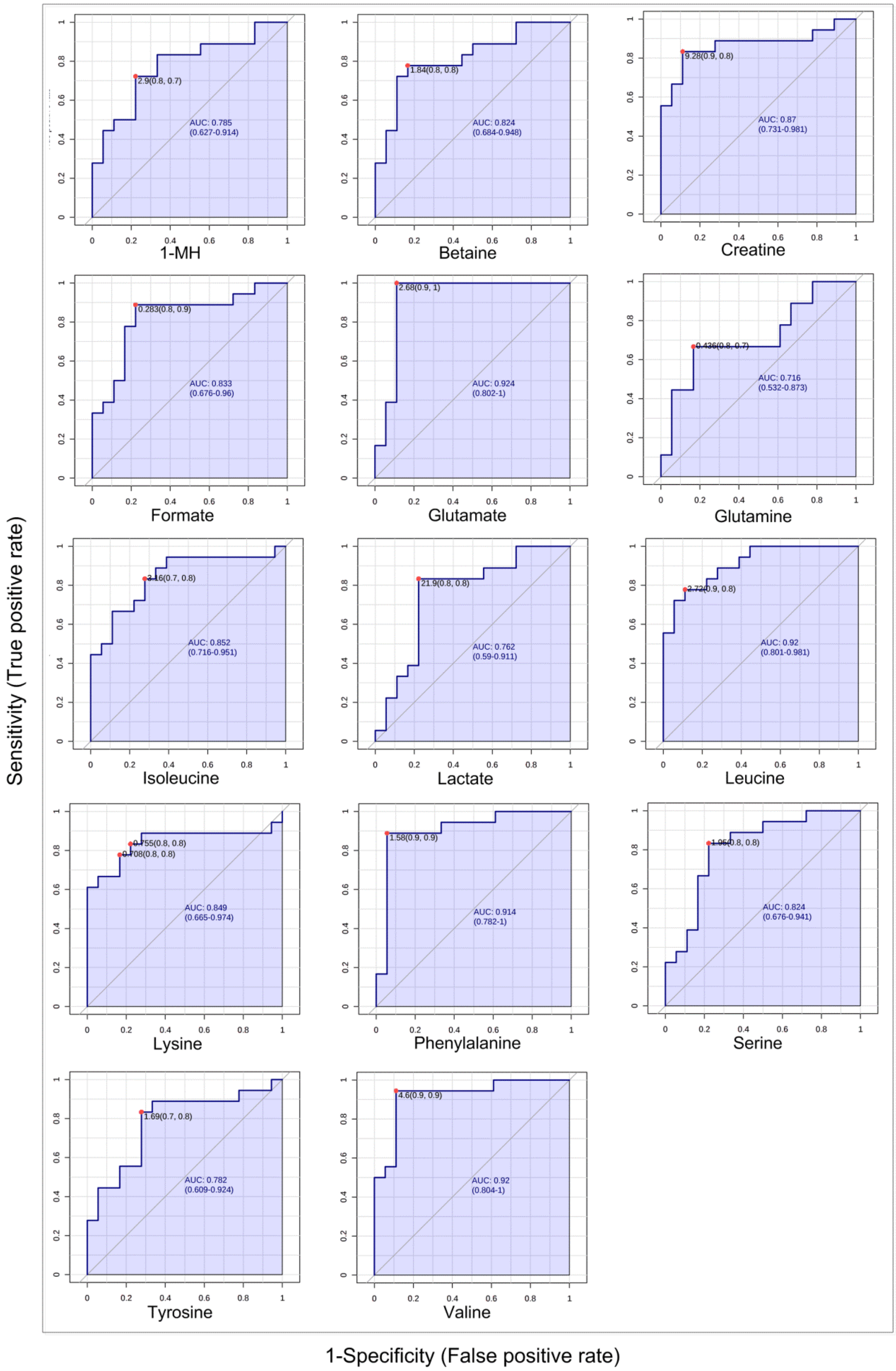

Additional file 4. Figure S4. Univariate ROC curve analyses of metabolites in serum for discrimination of HAE patients from healthy individuals.
