# Supplementary material for: Deciphering the metabolic perturbation in hepatic alveolar echinococcosis: a 1H NMR-based metabolomics study
Source: Parasit Vectors. 2019 Jun 13;12:300. doi: 10.1186/s13071-019-3554-0 (PMC6567409; doi:10.1186/s13071-019-3554-0)
Supplement: Supplementary file 6 — Additional file 6: Figure S6. PLS-DA scores plot (left panel) and permutation test (right panel) of PLS-DA model comprising 21 identified characteristic metabolites. [file 13071_2019_3554_MOESM6_ESM.pdf]

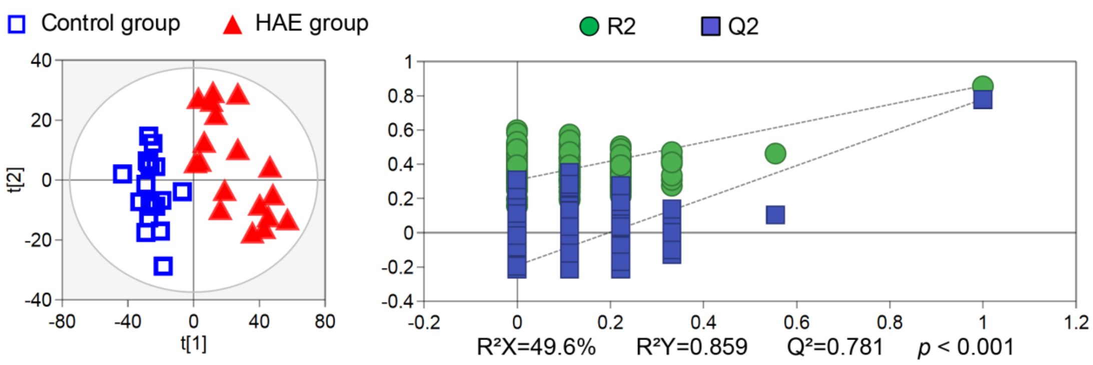

Additional file 6. Figure S6. PLS-DA scores plot (left panel) and permutation test (right panel) of PLS-DA model comprising 21 identified characteristic metabolites.
